# Elasticity of Carrier Fluid: A Key Factor Affecting Mechanical Phenotyping in Deformability Cytometry

**DOI:** 10.3390/mi15070822

**Published:** 2024-06-25

**Authors:** Hassan Pouraria, Jessica P. Houston

**Affiliations:** Department of Chemical and Materials Engineering, New Mexico State University, Las Cruces, NM 88003, USA; pouraria1@gmail.com

**Keywords:** deformability cytometry, microfluidics, elasticity, carrier fluid

## Abstract

Recently, microfluidics deformability cytometry has emerged as a powerful tool for high-throughput mechanical phenotyping of large populations of cells. These methods characterize cells by their mechanical fingerprints by exerting hydrodynamic forces and monitoring the resulting deformation. These devices have shown great promise for label-free cytometry, yet there is a critical need to improve their accuracy and reconcile any discrepancies with other methods, such as atomic force microscopy. In this study, we employ computational fluid dynamics simulations and uncover how the elasticity of frequently used carrier fluids, such as methylcellulose dissolved in phosphate-buffered saline, is significantly influential to the resulting cellular deformation. We conducted CFD simulations conventionally used within the deformability cytometry field, which neglect fluid elasticity. Subsequently, we incorporated a more comprehensive model that simulates the viscoelastic nature of the carrier fluid. A comparison of the predicted stresses between these two approaches underscores the significance of the emerging elastic stresses in addition to the well-recognized viscous stresses along the channel. Furthermore, we utilize a two-phase flow model to predict the deformation of a promyelocyte (i.e., HL-60 cell type) within a hydrodynamic constriction channel. The obtained results highlight a substantial impact of the elasticity of carrier fluid on cellular deformation and raise questions about the accuracy of mechanical property estimates derived by neglecting elastic stresses.

## 1. Introduction

Single-cell, high-throughput analyses generally involve the labeling of molecular biomarkers with exogenous fluorophores, many protocols of which require cell preparation, calibration, and standardization protocols before clinical diagnosis. Accordingly, emerging technologies are now focusing on how mechanical properties, akin to cellular antigens and proteins, are reporters of cellular function and disease. Notably, alterations in cellular mechanical properties have for decades been correlated to cellular function owing to the characterization of cytoskeletal and nuclear organization [1,2]. In general, mechanical traits, often dubbed “mechanical biomarkers”, encompass the deformability of cells when subjected to external loads. Comparable to the intrinsic properties, such as gene and protein expression, these mechanical biomarkers contribute to the phenotyping of cell populations. Several studies have established connections between alteration in cellular mechanical properties and various processes, including cell cycle progression [3], cancer malignancy [4,5,6], leukocyte activation [7,8], and stem cell differentiation [9,10]. Hence, by assessing cell mechanics, the requirement for external markers, such as fluorescent dyes, is eliminated, making it a compelling and noninvasive biomarker for cell identification.

Conventional methodologies for mechanical phenotyping of cells include optical stretching [11], atomic force microscopy [12], micropipette aspiration [13], and parallel plate rheology [14]. Such methods evaluate the response of the cell under the applied forces and estimate the mechanical properties of the cells, such as stiffness and viscous modulus. Despite their reliable results, these approaches suffer from a considerable drawback, which is low throughput, thus limiting their applicability for characterizing large populations of cells.

Accordingly, microfluidically driven cellular deformation approaches have become prominent owing to their high-throughput capabilities. In general, there are two main types of microfluidic deformation methods. The first method—constriction deformable cytometry—involves the use of a constriction channel narrower than the cell size [15,16]. Cells pass through the constriction channel, and their mechanical properties are evaluated based on passage times. The second approach—hydrodynamic forced deformation—employs either a constriction channel slightly larger than the size of cells [3] or a cross-slot channel generating a high extensional flow region [17]. Typical throughputs for constriction deformability, hydrodynamic constriction deformability, and hydrodynamic cross-slot deformability are 1, 100, and 1000 cells per second, respectively [3,18]. The hydrodynamic constriction channel, which allows real-time deformability cytometry (RT-DC), is compatible with active sorting devices [19,20]. Operating at a low Reynolds numbers (Re < 1), generally, inertia is neglected in RT-DC devices [3]. Furthermore, using a narrow channel slightly larger than the cells, very high shear rates and stress intensities can be achieved while allowing for contactless deformation. Significant effort has been devoted to developing a theoretical framework for extracting the mechanical properties of cells [21,22,23]. Deformability cytometry using cross-slot geometry typically operates at higher throughputs, where inertial effects come into play. The emerging inertial forces can be leveraged for the pre-alignment of particles, ensuring that cells experience identical path lines and stresses. 

These microfluidic devices not only employ different channel geometries, but also utilize different probing time scales [18]. Generally, shortened measurement times are associated with rapid force application, which can significantly influence the response of the cell. Previous studies indicate that the high strain rate in cross-slot deformability cytometry can fail to detect responses to actin cytoskeleton perturbations [17]. However, such changes could be detected by using cross-slot geometries at lower flow rates [24,25]. 

For inducing sufficiently high hydrodynamic forces in the hydrodynamic constriction channel, high-viscosity fluids are employed [3,18]. However, in the cross-slot channel, mechanical phenotyping can be performed using both high- and low-viscosity fluids [24,25]. A commonly used high-viscosity fluid in such devices is methylcellulose dissolved in phosphate-buffered saline (MC-PBS) [3,18,25]. Due to the biocompatibility of methylcellulose and the capability of increasing the viscosity of carrier fluid, MC-PBS is widely used in both hydrodynamic constriction channels and cross-slot deformation cytometry. The calculation of the exerted hydrodynamic stresses in these devices hinges on the flow field and measured fluid viscosity, considering the fluid as a generalized Newtonian fluid (GNF) with shear thinning behavior. In fact, it is assumed that the observed deformation of the cell is mainly due to the applied shear stress from MC-PBS [22,23,26]. Nonetheless, polymer solutions, such as MC-PBS, usually exhibit viscoelastic behavior, and their elastic properties may lead to significant changes in fluid flow. A recent rheological investigation of MC-PBS solutions with different concentrations revealed the elasticity of these solutions [27]. Hence, it is crucial to investigate the behavior of MC-PBS solutions when flowing in the hydrodynamic constriction channel, especially in the regions close to the deforming cells where the fluid flow exhibits complex patterns with mixed kinematics. Therefore, disregarding the viscoelastic nature of carrier fluid may lead to significant errors in estimating the exerted stresses on the cells. Since cell stiffness is proportionate to the exerted stresses for a given deformation during experiments, neglecting a portion of exerted stresses could lead to a wrong estimation of cell stiffness. It is worth mentioning that the estimated cell stiffness in hydrodynamic constriction cytometry typically shows a discrepancy when compared to the measured results by using AFM [18,28]. It should be mentioned that, due to the distinct probing methods and operating conditions, individual methods for measuring the cell’s mechanical properties often yield significantly different results. This disparity was highlighted in a recent work that compared elastic and viscous moduli obtained through an array of methods—atomic force microscopy, magnetic twisting, particle tracking microrheology, parallel-plate rheometry, and optical stretching—for the same cell line maintained in the standardized condition [29]. The resulting values exhibited a range spanning two orders of magnitude for an elastic modulus and three orders of magnitude for a viscous modulus. These disparities are attributed to the differences in the applied stress and strain rates, probe size, probing lengths scale, and time scale, and whether cells are attached to a surface or suspended in a fluid [28]. Hence, it is imperative to comprehend the temporal and spatial distributions of stress/strain applied to the cells in each method.

To investigate the significance of incorporating the complete viscoelastic behavior of the MC-PBS solution, we conduct computational fluid dynamics (CFD) simulations of single-phase MC-PBS flow within a microchannel with dimensions similar to those used in hydrodynamic constriction cytometry. In parallel, simulations are conducted for MC-PBS flow utilizing a GNF model that accounts for shear thinning behavior, but disregards the elasticity of fluid. A comparative analysis of the predicted flow fields employing a viscoelastic model and GNF model is presented. Subsequently, we model the deformation of a deforming particle representing a promyeloblast (i.e., HL-60 cell) navigating through the hydrodynamic constriction microchannel. Two MC-PBS fluid models are employed: one incorporating fluid elasticity and the other excluding it. A comparative evaluation of the flow fields, generated stress, and predicted shape of the suspended cell in these two fluid models elucidates the impact of fluid elasticity on the cell’s deformation within the microchannel.

## 2. Numerical Method

Numerical simulations were conducted to investigate the behavior of MC-PBS flows within a narrow microchannel, with dimensions typically employed in hydrodynamics constriction deformability cytometry. Two CFD models were employed to predict the flow field of the MC-PBS solution: a simplified GNF model and a viscoelastic fluid model. The GNF approach employs the Carreau–Yasuda model, which accounts for shear thinning behavior, but ignores the elasticity of fluid. In contrast, the viscoelastic fluid model incorporated the linear form of Phan–Thien–Tanner (PTT) model, which is suitable for polymer solutions [30]. This model considers both viscous and elastic stresses as well as the shear thinning effect, providing a more comprehensive representation of fluid behavior [30,31]. The results obtained from these two models will be compared to assess the reliability of employing the GNF approach. 

Governing equations of the flow field are fully resolved to predict the velocity, pressure, and stress distributions. The governing equations of isothermal incompressible fluid flow in the laminar regime are as follows:

(1) ∇·u=0

(2) $$\rho \left(\frac{\partial u}{\partial t}+u\cdot \nabla u\right)=-\nabla p+\nabla \cdot \tau$$

(3) $$\tau =\tau_{s}+\tau_{p}=\mu_{s}\left(\nabla u+\nabla u^{T}\right)+\tau_{P}$$

(4) $$\lambda \left[\frac{\partial \tau_{P}}{\partial t}+\nabla \cdot u\tau_{P}\right]+f\left(Tr\tau_{P}\right)\tau_{P}=\mu_{p}\left(\nabla u+\nabla u^{T}\right)+\lambda \left(\tau_{P}\cdot \nabla u+\nabla u^{T}\cdot \tau_{P}\right)$$

(5) $$f\left(Tr\tau_{P}\right)=1+\frac{\lambda \varepsilon }{\mu_{p}}\;Tr\left(\tau_{P}\right)$$

**Figure 1:**
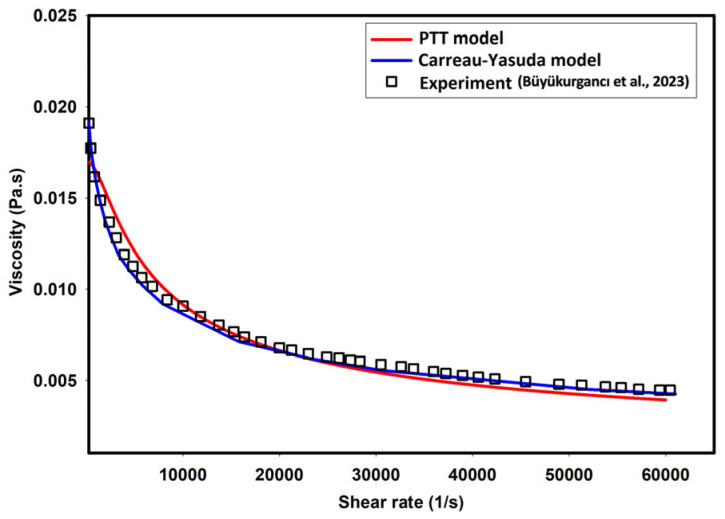
Experimental shear viscosity vs. PTT and Carreau–Yasuda models [27].

Equations (1) and (2) are continuity and momentum equations, respectively, where *u* represents the velocity, *p* is pressure, and τ indicates the stress tensor. In the viscoelastic fluid context, the stress tensor τ is the sum of solvent stress $(\tau_{s}$) and polymer stress ($\tau_{p}$) (Equation (3)). Polymer stress was modeled using the PTT model (Equations (4) and (5)), where λ and $\mu_{p}$ represent the polymer relaxation time and polymer viscosity, respectively, and ε is a material parameter related to its extensional property. Generally, ε may range from 0 to 1, and a lower ε value in the PTT model corresponds to a higher predicted uniaxial extensional viscosity. In fact, when ε tends to zero, the PTT model reduces to the well-known Oldroyd-B model. However, contrary to the Oldroyd-B model, the PTT model predicts the shear-thinning behavior of fluid when ε≠0. The shear viscosity function in the PTT model is obtained by determining the appropriate value of ε [32,33]. On the contrary to the viscoelastic models, in the context of the GNF approach, the stress tensor includes only the first term on the right-hand side of Equation (3), representing the shear stress. The total shear viscosity, μ, is modeled using the Carreau–Yasuda model that follows Equation (6).

(6) $$\mu \left(\dot{\gamma }\right)=\mu_{\infty }+\left(\mu_{0}-\mu_{\infty }\right)\cdot {\left(1+{\left(\lambda \dot{\gamma }\right)}^{\alpha }\right)}^{\frac{v-1}{\alpha }}$$

where $\mu_{0}$ and $\mu_{\infty }$ are zero shear and infinite shear viscosities, respectively. Furthermore, v, λ, and α are the power law index, relaxation time, and Carreau–Yasuda model constant, respectively. CFD simulations were conducted for the 0.5% MC-PBS solution. Experimental data regarding measured viscosity were obtained from Büyükurgancı et al. [27], where concentric cylinder (CC), cone–plate (CP), plate–plate (PP), and parallel-disk (PP) measurements were performed to cover very low to high shear rates. The viscosity of the solvent was assumed to be 1 mPa·S. Table 1 shows the parameters used to describe the viscosity of the 0.5% MC-PBS solution using the Carreau–Yasuda model [27]. Furthermore, Figure 1 compares the viscosity of the 0.5% MC-PBS solution as predicted by the PTT model (ε=0.013) and Carreau–Yasuda model with the experimental measurements. As depicted, the PTT and Carreau–Yasuda models reasonably captured the shear thinning behavior of the fluid observed in the experimental data [27]. 

Numerical modeling was initially conducted to analyze a single-phase flow of the 0.5% MC-PBS solution within a 3D square channel. The primary aim was to compare the predicted stresses utilizing the Carreau–Yasuda model and the more comprehensive viscoelastic model know as PTT. Additionally, CFD simulations were performed to replicate the deformation of an HL-60 cell suspended in 0.5% MC-PBS solution when passing the channel. To this end, a viscous droplet model was employed, which takes into account the apparent cytoplasmic viscosity and membrane tension of cells. We used the level set method to model the two-phase flow and track the interface of the primary phase (0.5% MC-PBS solution) flowing in the microchannel and a suspended droplet representing the cell. As such, the following equation was solved in conjunction with Equations (1)–(5):

(7) $$\frac{\partial \emptyset }{\partial t}+u\cdot \nabla \emptyset =0$$

For the solution, volume fraction (α) and local material properties are updated based on ∅ and smoothed across the interface using a smooth Heaviside function. Furthermore, surface tension force is added to the right-hand side of the momentum equation and calculated as follows:

(8) $$F_{s}=\sigma kn\delta_{s}$$

where σ represents the surface tension coefficient, *k* denotes the interface curvature, *n* signifies the normal vector, and $\delta_{s}$ represents the smoothed Dirac delta function centered at the interface. To restore its correct distributions near the interface, a re-distancing problem was solved [34,35,36]. 

Transport equations were solved by using the finite volume method (FVM) with the TransAT 5.7 code [37]. In the CFD model, pressure–velocity coupling was achieved by using the SIMPLEC algorithm. The spatial derivatives were discretized using a 2nd-order hybrid linear/parabolic approximation (HLPA) scheme. For the level set model, we used a 3rd-order WENO scheme for re-distancing [38]. The residual levels for convergence criteria were set to 1 × 10^−6^ for the velocity, level set, and stress components, and 1 × 10^−5^ for pressure.

## 3. Results and Discussions

### 3.1. Flow Field in the Microchannel Conveying the Single-Phase MC-PBS Solution

**Figure 2:**
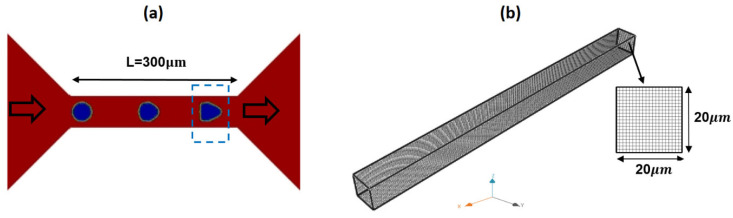
A schematic of typical microchannel geometry in hydrodynamic constriction deformability cytometry (**a**) and the adopted 3D grid in the CFD model (**b**).

Numerical simulations were performed to analyze the flow characteristics of the 0.5% MC-PBS solution within a microchannel commonly utilized in hydrodynamic constriction deformability cytometry [18]. CFD simulations were initially employed to study the single-phase flow of the carrier fluid, utilizing two rheological models: the Carreau–Yasuda model and PTT model. Figure 2 shows a schematic of the microchannel and the adopted geometry and dimensions in the CFD model. The width, height, and length of the channel are 20 µm, 20 µm, and 400 µm, respectively. The dimensions of the microchannel and flow rate are identical to those reported experimentally [18]. A uniform grid size of 1 μm (total cell = 160,000) was adopted in the 3D CFD simulations. 

**Figure 3:**
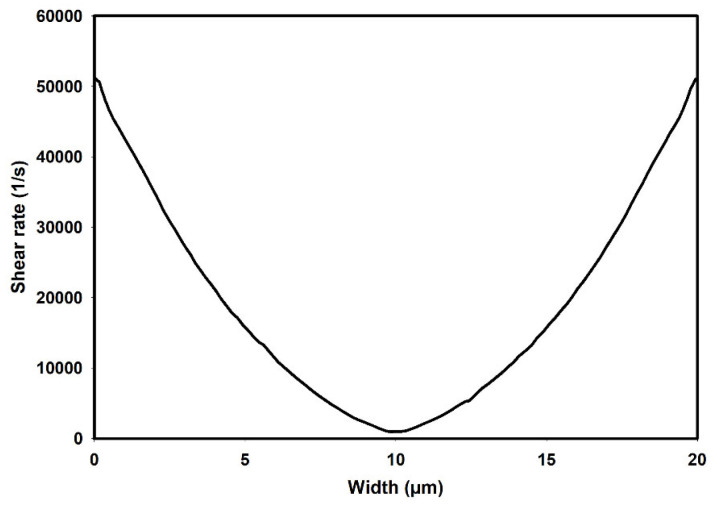
Shear rate distribution across the width of the channel as predicted by the Carreau–Yasuda model.

Figure 3 illustrates the shear rate distribution as predicted by the Carreau–Yasuda model along the width of the channel, corresponding to an average flow velocity of 0.1 m/s under fully developed flow conditions. As observed, the predicted shear rates vary from nearly zero at the center of the channel to a maximum value of approximately 52,000 1/s at the walls. 

According to Figure 1, both the Carreau–Yasuda and PTT models can reasonably predict the shear viscosity of carrier fluid in the predicted shear range. 

**Figure 4:**
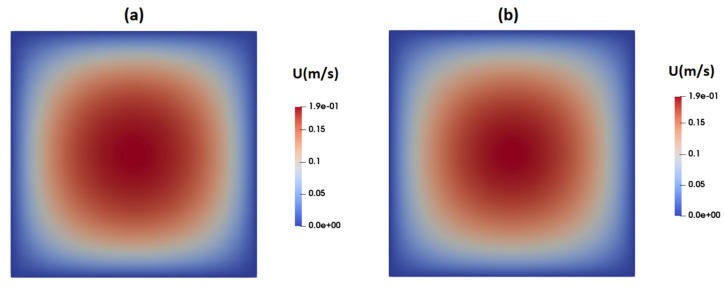
Distribution of axial velocity as predicted by (**a**) the PTT model and (**b**) Carreau–Yasuda model.

Figure 4a,b present contours illustrating the axial velocity within the cross-section of the channel, as predicted by the Carreau–Yasuda and PTT models upon reaching fully developed flow conditions. Notably, both models yield remarkably similar velocity distributions, showcasing a peak velocity of approximately 0.19 m/s at the channel center. In general, when dealing with shear-thinning fluids, one would anticipate flattened velocity profiles in comparison to their Newtonian counterparts. 

**Figure 5:**
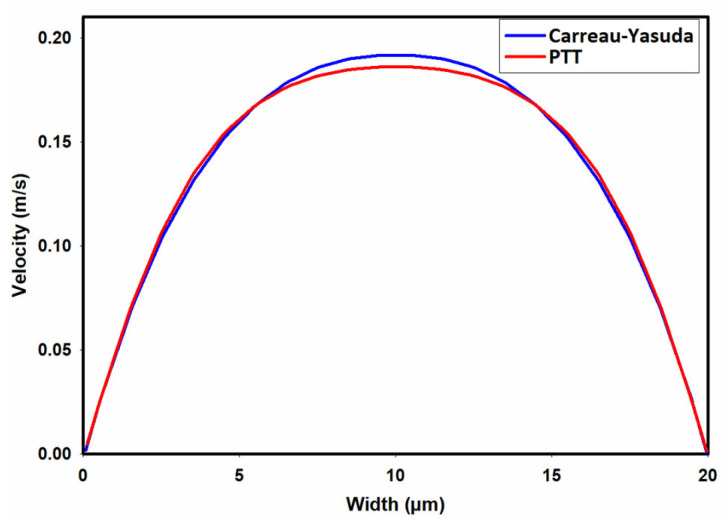
Velocity distribution across the width of the channel.

Figure 5 provides a more discernible representation of the contrast in axial velocity distribution across the channel width as determined by the PTT and Carreau–Yasuda models. As depicted in this figure, the PTT model predicts a slightly more flattened velocity profile at the center of the channel, suggesting slightly elevated shear-thinning behavior. The discrepancies observed in these velocity profiles could be attributed to the prediction of slightly different viscosities by the PTT and Carreau–Yasuda models, especially at high shear rates, as shown in Figure 1.

**Figure 6:**
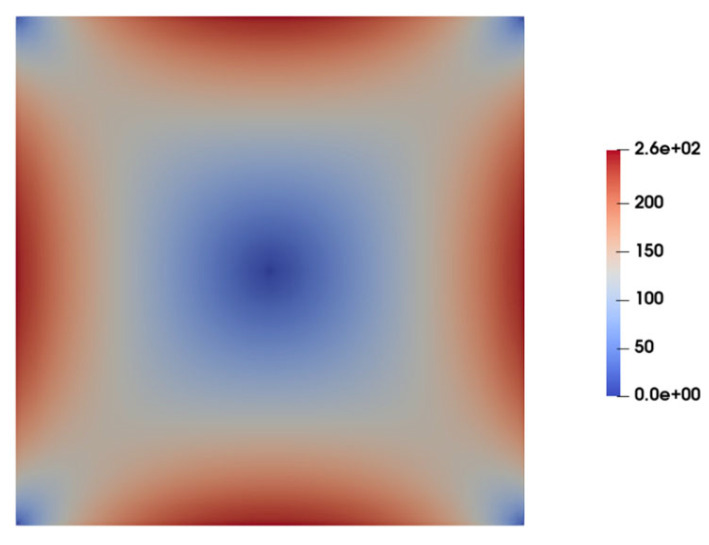
Contours of total shear stress at the channel cross-section as predicted by the Carreau–Yasuda model.

**Figure 7:**
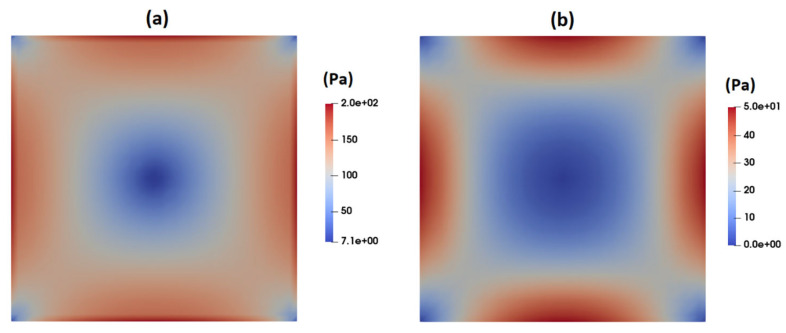
Contours of shear stress at the channel cross-section as predicted by the PTT model: (**a**) polymer shear stress and (**b**) solvent shear stress.

**Figure 8:**
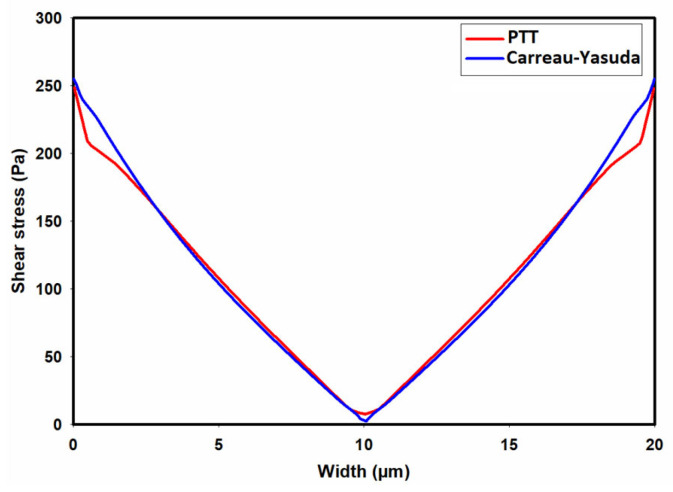
Distribution of total shear stress ($\tau_{xy}$) across the width of the channel as predicted by the PTT and Carreau–Yasuda models.

Figure 6 illustrates the contours of total shear stress as predicted by the Carreau–Yasuda model at the cross-section of the channel under fully developed flow conditions. As observed, the shear stress is at its maximum at the walls and gradually decreases to nearly zero when moving toward the center of the channel, where the velocity gradient is zero. In contrast to the Carreau–Yasuda model, the PTT model directly accounts for the contribution of polymers in the stress tensor. That is, the total stress is calculated as a summation of solvent and polymer stresses. Figure 7a,b display the contours of polymer shear stress and solvent shear stress as predicted by the PTT model within the channel cross-section. Figure 8 illustrates the predicted total shear stress obtained by the PTT model and Carreau–Yasuda model across the width of the channel. As observed in this figure, there is good agreement between the predicted total shear stresses by the two models, except in regions close to the walls where the PTT model predicts slightly lower shear stresses when compared to the Carreau–Yasuda model. This small difference can be attributed to the accuracy of fitting the experimental shear viscosities by these two models. 

**Figure 9:**
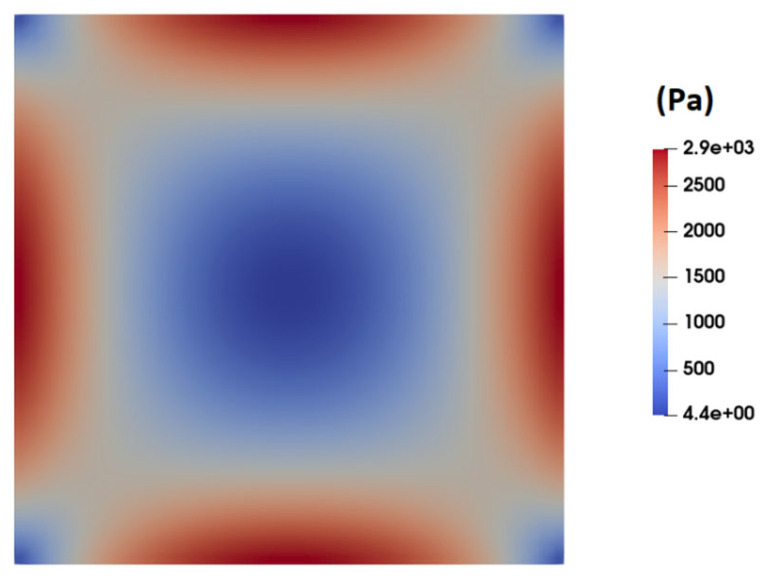
Distribution of normal stress ($\tau_{XX})$ at the channel cross-section as predicted by the PTT model.

**Figure 10:**
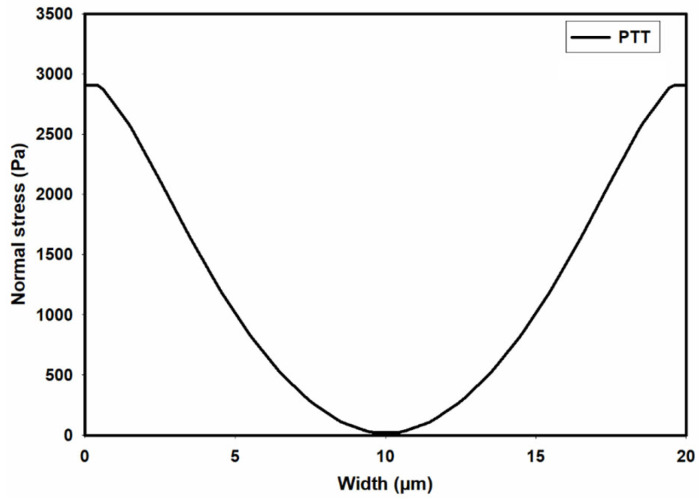
Distribution of normal stress ($\tau_{xx})$ across the width of the channel as predicted by the PTT model.

Figure 9 shows the contour of normal stress ($\tau_{xx})$ at the channel cross-section as predicted by the PTT model. This figure clearly shows significantly high elastic stress in the proximity of the channel walls, gradually diminishing to nearly zero at the channel center. Figure 10 presents the distribution of normal stress ($\tau_{xx})$ across the channel width. A comparison of the predicted normal stress ($\tau_{xx})$ in Figure 10 with the total shear stress in Figure 8 unequivocally reveals that the normal stress magnitude within the channel is an order of magnitude higher than that of the total shear stress, as predicted by the PTT and Carreau–Yasuda models. Previous studies underscore a substantial influence of such normal stresses on flow fields containing suspended particles. In fact, the observed stress gradients possess the potential to significantly influence the trajectory of suspended particles or induce deformations in the shape of pliable particles navigating through such flow fields [39,40,41,42,43].

**Figure 11:**
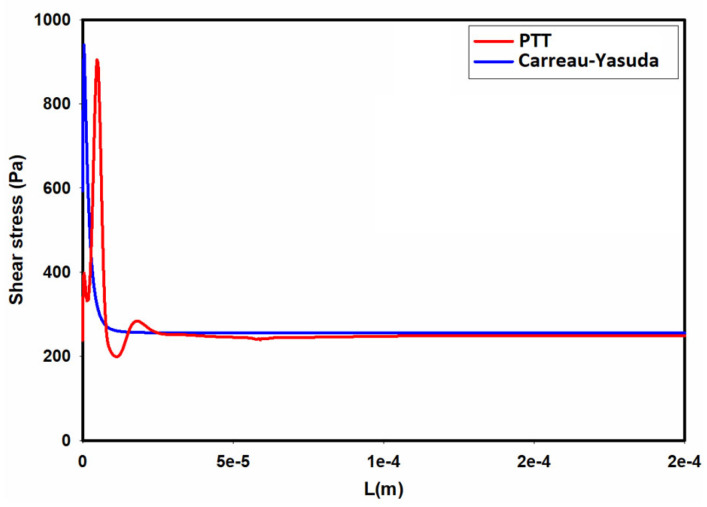
Distribution of average shear stress at the wall along the channel as predicted by the PTT and Carreau–Yasuda models.

While the Carreau–Yasuda model can reasonably predict the velocity profile and total shear stresses in steady shear flows, it completely neglects the generation of normal stresses. Furthermore, its inability of directly accounting for the contribution of polymer chains in the stress tensor can be problematic in unsteady flows and flows with mixed kinematics [44,45]. Figure 11 shows the distribution of average shear stress at the wall of the channel as we move from the inlet toward the outlet. As seen in Figure 11, the predicted shear stresses by using the Carreau–Yasuda and PTT models in the developing region are different. According to this figure, for a single-phase flow of the 0.5% MC-PBS solution in a square channel, the flow reaches a fully developed condition within a short length in the entrance region. Hence, overall, using the Carreau–Yasuda model results in a reasonable estimation of the shear stress distribution in the channel. 

### 3.2. Numerical Modeling of Cell Deformation in a Hydrodynamic Constriction Channel

The preceding numerical results pertaining to the single-phase flow of the 0.5% MC-PBS solution in the microchannel underscore the prevalence of normal stress over shear stress. In this section, we employ the level set model to investigate the impact of carrier fluid’s elasticity on the exerted stress and deformation of suspended particles. In fact, due to the large size of the cells compared to the size of the channel, the flow field is substantially altered as the cells move within the channel. Hence, we model the deformation of a suspended viscous particle, representing an HL-60 cell, navigating through a hydrodynamic constriction microchannel using two MC-PBS fluid models—one incorporating elasticity and the other lacking it.

A method employed for modeling cell deformation is the viscous droplet model, where the cell is conceptualized as a viscous fluid-filled bag with constant surface tension. This model takes into account apparent membrane tension and cytoplasmic viscosity. While not appropriate for all cell types, it has demonstrated reasonable accuracy in describing the deformation of HL-60 cells [13,46,47]. It is crucial to emphasize that the main objective of this study is to explore the significance of the emerging elastic stress surrounding cells in motion within the channel and to investigate whether this elastic stress plays a crucial role in cell deformation compared to viscous stress. Consequently, irrespective of the accuracy of the viscous droplet model in replicating the exact behavior of HL-60 cells, any discernible differences in deformation predicted by two CFD models—one including the elastic effect and the other excluding it—would underscore the pivotal role of fluid elasticity in cell deformation.

**Figure 12:**
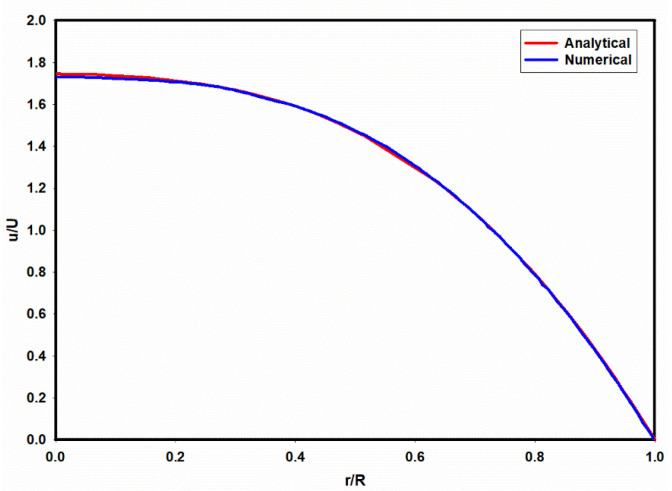
A comparison of the predicted velocity profiles using analytical and numerical models. De = 6.3, β = 0.1, and ε=0.25.

We model the deformation of an HL-60 cell under an operating condition reported experimentally [18]. In order to reduce the computational cost, an axisymmetric CFD model is employed, which is compatible with the observed cell shape in the experiments. Hence, the predicted CFD results show the deformation of a single cell flowing in a tube with an identical hydraulic diameter (20 µm) rather than a 3D square channel. The accuracy of the CFD model in predicting the velocity profile of viscoelastic fluid within a circular tube was first validated by comparing the predicted results using the PTT model with an analytical solution [32]. Figure 12 shows the predicted velocity profile for a viscoelastic fluid, where the solvent viscosity ratio (β), Deborah number (De), and extensibility parameter (ε) are 0.1, 6.3, and 0.25, respectively. As observed in Figure 12, there is good agreement between the predicted numerical results and the analytical solution.

Subsequently, CFD simulations were conducted for a two-phase flow of a suspended viscous droplet in the 0.5% MC-PBS solution and for a mean flow velocity of 0.1 m/s. Previous studies revealed that the apparent viscosity of HL-60 cells depends on the imposed shear rate. Using the empirical model proposed by Tsai et al. and considering an average shear rate of 20,000 1/s in the channel, the apparent viscosity was estimated to be 1.08 Pa·s [48]. Furthermore, the cortical membrane tension and density of HL-60 are 155 pN/μm and 1080 Kg/m^3^, respectively [47,49]. The droplet representing an HL-60 cell was assumed to be initially spherical with a diameter of 14 μm at the inlet. Simulations were conducted using quad grids with a size of 0.5 μm (20 × 800 cells). The transient simulation was continued until the droplet traveled 300 μm downstream from the inlet. This distance mirrors the cell’s journey to the measurement point within the lengths of the hydrodynamic constriction channel [18]. Furthermore, adaptive time stepping was employed to ensure that the Courant number remained below 0.25 in the solution. 

**Figure 13:**
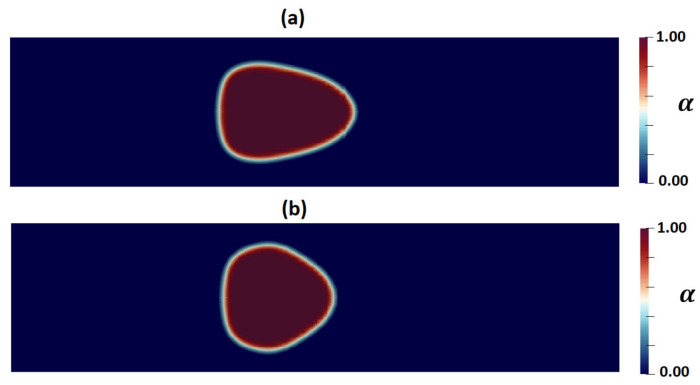
Contours of cell volume fraction (α) as predicted by (**a**) the PTT and (**b**) Carreau–Yasuda models.

**Figure 14:**
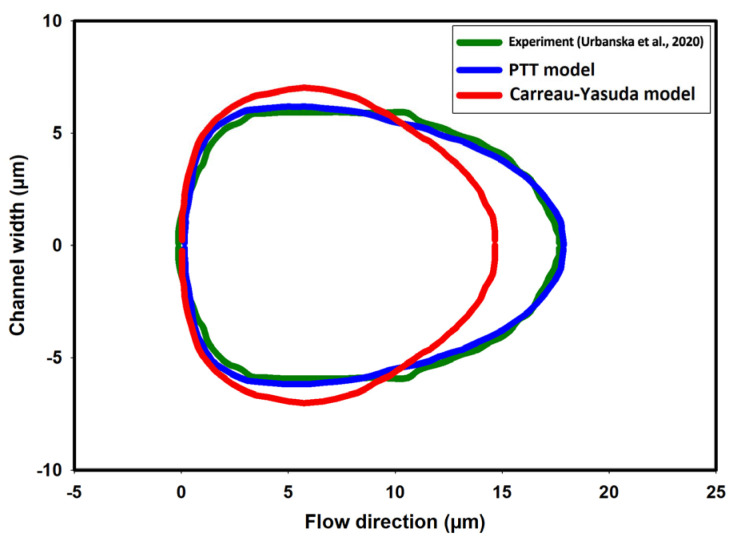
A comparison of cell deformations as measured in the experiment and predicted by the PTT and Carreau–Yasuda models [18].

Figure 13a,b show the shape of droplets 300 mm downstream from the inlet as predicted by the PTT and Carreau–Yasuda models, respectively. A discernible contrast in droplet deformation is evident, with the PTT model indicating greater deformation due to the consideration of fluid elasticity. Figure 14 compares the cell shape predicted by CFD simulations using the PTT and Carreau–Yasuda models with experimental data on cell shape. Aside from demonstrating a discernible difference in the predicted deformation between the two CFD models, the obtained results exhibit an excellent agreement between the predicted shape using the viscoelastic fluid model and the experimental observation under the specific experimental condition used in this study. However, employing more sophisticated cell models would result in more reliable predictions for different cells and across various time scales [26,50].

**Figure 15:**
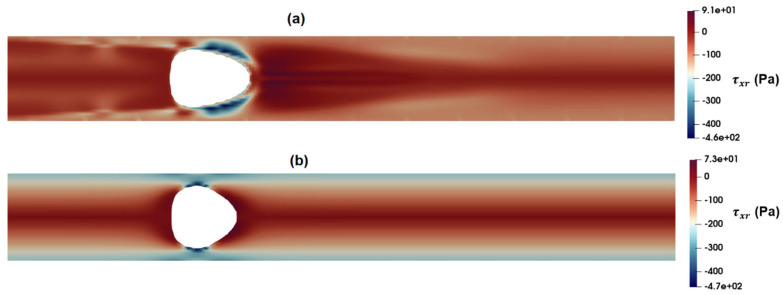
Distribution of shear stress as predicted by the (**a**) PTT and (**b**) Carreau–Yasuda models.

Figure 15 shows the distribution of shear stress around the suspended droplets 300 μm downstream of the inlet as predicted by the PTT (Figure 15a) and Carreau–Yasuda (Figure 15b) models. According to these figures, the maximum shear stress near the droplet in the Carreau–Yasuda model is higher than that predicted by the PTT model. Moreover, the overall predicted distribution of shear stress is significantly different between the two. 

**Figure 16:**
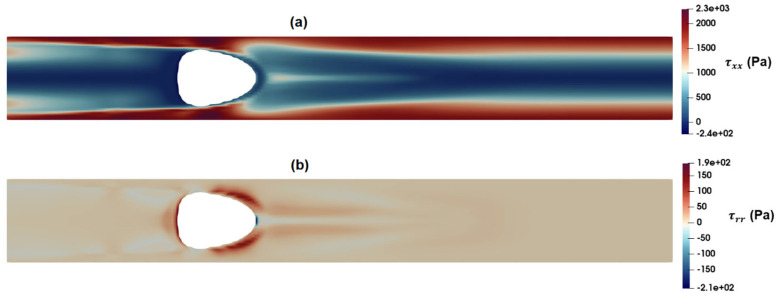
Distribution of normal stress components as predicted by the PTT model: (**a**) $\tau_{xx}$ and (**b**) $\tau_{rr}$.

Figure 16a,b show the distribution of axial and radial normal stresses around the suspended droplets. A comparison of these figures with Figure 15a,b indicates that the intensity of the radial component of elastic stress, $\tau_{rr},$ is less than that of shear stress, while the axial component of normal stress, $\tau_{xx},$ reaches significantly high values around the droplet. According to the present results, the intensity of axial normal stress is significantly higher than that of shear stress around the droplet, which in turn results in exerting a compressive force and further stretching the deformable particle. 

**Figure 17:**
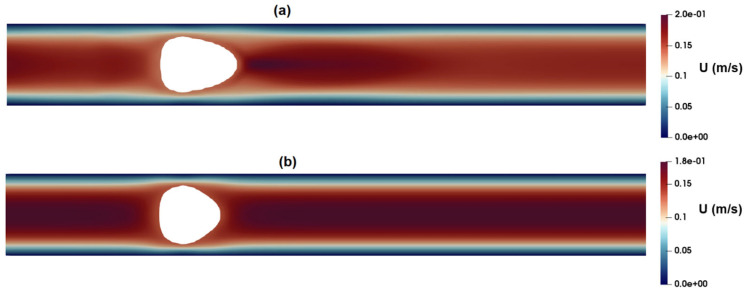
Distribution of axial velocity as predicted by the (**a**) PTT and (**b**) Carreau–Yasuda models.

**Figure 18:**
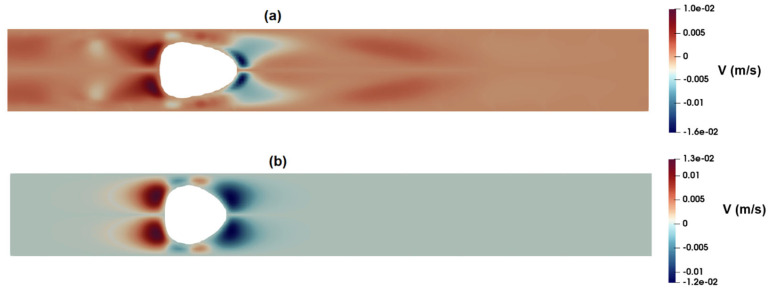
Distribution of radial velocity as predicted by the (**a**) PTT and (**b**) Carreau–Yasuda models.

Figure 17a,b show the distribution of the axial velocity around the suspended droplets as predicted by the PTT and Carreau–Yasuda models, respectively. As observed in these figures, the PTT model predicts a high-velocity region at the front of the droplet, while the velocity distribution predicted by the Carreau–Yasuda model shows a velocity profile similar to that of fully developed flow. Figure 18a,b show the distribution of radial velocity around the suspended droplets as predicted by the PTT (Figure 18a) and Carreau–Yasuda (Figure 18b) models. According to these figures, the radial velocity at the rear and front parts of droplet are toward the wall and center of the channel, respectively. Such velocity distributions suggest the formation of a contraction flow regime in the front region of the droplet. Apart from their inability to predict elastic stress and time dependency, GNF models, such as Carreau–Yasuda, also predict a constant Trouton ratio, that is the ratio of extensional viscosity to shear viscosity, which remains constant at the Newtonian value of 3. Thus, their application for flow fields with mixed kinematics is more limited [44]. Figure 16a and Figure 17a clearly show an extensional region predicted by the PTT model in the front region of the droplet, while the Carreau–Yasuda model fails to predict such an extensional flow. Moreover, the memory effect inherent in viscoelastic fluids necessitates a careful consideration of the minimum distance between two passing cells or the frequency of events. This ensures that the flow field at a specific point has adequate time to attain the equilibrium condition encountered by the proceeding cell. Without this attention to temporal and spatial dynamics, the observed deformation of distinct cells may not yield reliable results when estimating their mechanical properties or conducting comparative analysis.

## 4. Conclusions

The application of microfluidic systems for deformability cytometry has gained momentum, with numerous laboratories aiming to translate these technologies into commercially viable cytometers. Despite the common use of viscoelastic carrier fluids, the characterization of cells within these devices often relies on simplified computational frameworks that only account for viscous stresses while neglecting fluid elasticity.

The present study investigates the impact of the elasticity of the commonly used carrier fluid, the MC-PBS solution, on cell deformation in hydrodynamic constriction deformability cytometry. Three-dimensional computational analysis was conducted using two fluid models: a conventional GNF model that considers shear-thinning behavior but disregards fluid elasticity, and a comprehensive viscoelastic model, PTT, which incorporates both shear-thinning and elastic properties. The numerical results for the single-phase flow of a 0.5% MC-PBS solution indicate that both models yield nearly identical distributions of shear stress and velocity fields for fully developed steady shear flow. However, the incorporation of the viscoelastic model reveals significant normal stress generation along the channel, contradicting the assumption of zero normal stress in commonly used GNF fluid models. Importantly, CFD results highlight that the predicted intensity of normal stress surpasses that of shear stress.

The level set model was employed to predict the transient flow of suspended viscous particles representing HL-60 cells in a 0.5% MC-PBS solution. Initially neglecting fluid elasticity and subsequently considering the viscoelastic nature of the carrier fluid enabled a comprehensive comparison of predicted flow fields, stress distributions, and resulting deformations.

The obtained CFD results unveil substantial alterations in the flow field around moving particles within the channel. Accounting for the viscoelastic behavior of the carrier fluid led to slightly different predictions of shear stress distribution around the particles. However, the primary disparity lies in the prediction of significant normal stresses around the deforming particles, indicating a notable compressive force.

Comparing the predicted particle shapes using two CFD models—one incorporating elastic effects and the other excluding them—underscored the pivotal role of fluid elasticity in particle deformation. The predicted velocity fields around the moving particles also revealed a combination of shear and extensional flows in the polymer solution, necessitating the use of a CFD model that considers the viscoelastic behavior of the fluid. 

These findings raise pertinent questions regarding the accuracy of mechanical property estimates derived from neglecting elastic stresses. The observed disparities underscore the importance of considering fluid elasticity in deformability cytometry analysis, particularly for chips with hydrodynamic constriction channels.

## Figures and Tables

**Table 1:** Carreau–Yasuda model constants.

Fluid	ϻ_0_	ϻ_∞_	λ	*v*	α
0.5% MC-PBS	20 mPa.s	1 mPa.s	0.0012	0.65	1.02

## Data Availability

The original contributions presented in the study are included in the article, further inquiries can be directed to the corresponding author.
